# Effects of High and Low Training Volume with the Nordic Hamstring Exercise on Hamstring Strength, Jump Height, and Sprint Performance in Female Football Players: A Randomised Trial

**DOI:** 10.1155/2022/7133928

**Published:** 2022-08-31

**Authors:** Roar Amundsen, Janita Sæther Heimland, Solveig Thorarinsdottir, Merete Møller, Roald Bahr

**Affiliations:** ^1^Department of Sports Medicine, Oslo Sports Trauma Research Center, Norwegian School of Sport Sciences, Oslo, Norway; ^2^Department of Sports Science and Clinical Biomechanics, University of Southern Denmark, Odense, Denmark

## Abstract

The evidence-based hamstring strengthening programme for prevention of hamstring injuries is not adopted by football teams because of its high training volume. This study on female football players investigated if high-volume training with the Nordic hamstring exercise is more effective on hamstring strength, jump height, and sprint performance than low-volume training. We also examined the time course of changes in muscle strength during the intervention period. Forty-five female football players were randomised to a high- (21 sessions, 538 total reps) or low-volume group (10 sessions, 144 total reps) and performed an 8-week training intervention with the Nordic hamstring exercise during the preseason. We tested hamstring strength (maximal eccentric force with NordBord and maximal eccentric torque with isokinetic dynamometer), jump height, and 40 m sprint before and after the intervention. The NordBord test was also performed during training weeks 4 and 6. Both groups increased maximal eccentric force (high-volume: 29 N (10%), 95% CI: 19–38 N, *p* < 0.001, low-volume: 37 N (13%), 95% CI: 18–55 N, *p* = 0.001), but there were no between-group differences (*p* = 0.38). Maximal eccentric torque, jump height, and sprint performance did not change. Maximal eccentric force increased from the pretest to week 6 (20 N (7%), 95% CI: 8 to 31 N, *p* < 0.001), but not week 4 (8 N (3%), 95% CI: −2 to 18 N, *p* = 0.22). High training volume with the Nordic hamstrings exercise did not lead to greater adaptations in strength, jump height, or speed than a low-volume programme. Players in both groups had to train for at least 6 weeks to improve maximal eccentric force significantly.

## 1. Background

Women's elite football has developed rapidly during the last decade. The level of play has improved [1], there are more professional players [2, 3], and they face higher training loads and competition demands [4, 5]. Each of these factors may have altered injury risk. Hamstring injury has been the most common injury in men's elite football for years [6,7] and has now also become one of the most common injury types for women [8, 9]. Preventing the most common injuries is essential for football teams and players, as they affect team performance [10, 11], player performance [12, 13], and player development [14].

Male football players performing a programme using Nordic hamstring exercise can more than halve the risk of hamstring injuries [15, 16], probably by increasing eccentric hamstring strength and muscle fascicle length [17, 18]. Female elite football players can also increase their eccentric strength and fascicle length with a preseason programme of the Nordic hamstring exercise [19]. However, the long duration (8–13 weeks) and high training volumes (2–3 sessions per week, up to 30 repetitions per session) of these programmes reduce the chance of implementation [20]. In men's elite football, fewer than one fifth of teams report being fully compliant with the full evidence-based hamstring injury prevention programme [21, 22].

A programme of shorter duration and with lower training volume can facilitate implementation in the busy training and match schedules of elite teams but may attenuate the effects as there is a dose-response relationship between strength gain and training volume [23]. Interestingly, recreationally active men performing a high training volume of the Nordic hamstring exercise did not improve their eccentric strength more than those performing a low training volume (440 vs. 128 total reps over 6 weeks), and the muscular adaptations for both groups occurred early during the intervention [24]. However, the training volumes needed to improve strength can be affected by training status [25], concurrent training [26], and sex [27], so the results from recreationally active men may not be transferable to female football players.

Therefore, we conducted a training intervention with female football players where the primary aim was to determine if using the evidence-based high-volume programme of the Nordic hamstring exercise was more effective on hamstring strength, jump height, and speed than a low-volume programme. We also aimed to examine the time-course of changes in muscle strength during the 8-week intervention period and to compare the results on eccentric strength when assessed by two common testing devices, a Nordic hamstring testing device, and an isokinetic dynamometer.

## 2. Materials and Methods

### 2.1. Trial Design and Participants

We invited 45 players (21 ± 4 yrs, 169 ± 6 cm, 63 ± 8 kg) from two Norwegian 2nd tier women's football teams to participate in this randomised trial during their preseason period (Jan–Mar 2021). Both teams had 5–7 football training sessions and played one training match per week. The Norwegian Center for Research Data and the Norwegian School of Sports Sciences' Ethics Committee approved the study. All players included were members of the first team squads, above 16 years old, and gave their individual informed consent to participate.

### 2.2. Training Intervention

**Figure 1:**
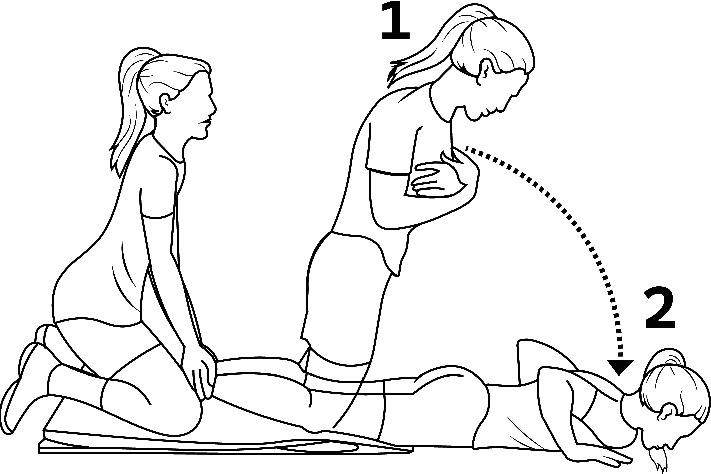
Illustration of how the Nordic hamstring exercise was performed.

We randomised players within teams to a high- or low-volume group. Training prescriptions were based on previous studies [15, 24, 28] but adjusted to an 8-week intervention period to match the preseason (Table 1). Players performed the Nordic hamstring exercise in pairs after football training sessions (Figure 1). One player knelt on both knees, crossed the arms on her chest, had the partner hold her ankles, and then leaned slowly forward without flexing the hip while using her hamstrings to resist the falling motion for as long as she could. Players used their arms to buffer the fall and push themselves back up to the starting position. If players were able to control the fall throughout the range of motion, load was increased by adding speed to the starting phase of the motion [28]. Verbal encouragement was given during all repetitions to ensure maximal effort. Before every Nordic hamstrings session, players reported the maximal hamstring muscle soreness felt since the previous session on a numerical rating scale (0 = no pain, 1–3 = mild, 4–6 = moderate, 7–9 = severe, and 10 = worst pain imaginable). A researcher attended all Nordic hamstring training sessions to ensure high compliance, proper execution of the exercise and prevent contamination between groups. We aimed to have at least 48 h between Nordic hamstring sessions, but to adjust for the team training plan and match schedule only 24 h separated some sessions in the high-volume group.

### 2.3. Testing Procedures and Outcomes

The pre- and posttests were conducted at the Norwegian School of Sports Sciences. Players performed a 15 min standardised warm-up led by a researcher, with cycling, running drills, and active stretching.

Maximal eccentric hamstring force was tested in a Nordic hamstring testing device [29] (NordBord v. 1.0, VALD Performance, Albion, Australia). Players knelt on the board and had their ankles secured by ankle hooks attached to uniaxial load cells. We instructed them to cross their arms in front of their chest, move slowly forward without bending their hip, and resist the forward falling motion for as long as possible. Players performed three submaximal warm-up repetitions with a subsequent 2 min break, before three sets with maximal effort consisting of three repetitions with no added weight, one repetition with a 5 kg weight vest and one repetition with a 10 kg weight vest. Verbal encouragement was given during the test to ensure maximal effort from the players. The three maximal sets were separated with 1 min breaks. The sets with added weight were included to ensure that all players reached a “breaking point,” where they were unable to control the forward falling motion. We recorded the maximal force (N) produced in the right and left leg for each of the three sets, and the average from the right and left leg is reported.

Maximal isometric and eccentric knee flexor torque were tested unilaterally, right leg before left, in an isokinetic dynamometer (Humac Norm model 502140, Computer Sports Medicine Inc., Stoughton, MA, USA). Players were seated with 90 degrees hip flexion and the dynamometer aligned with the knee joint axis. Straps were fixed around the hip, shoulders, and the thigh to minimise other movement. After a standardised warm-up with four isokinetic concentric repetitions of knee flexion (60 degrees/s) and 30 s rest, players performed isometric tests with the knee 90 degrees, 60 degrees, and 30 degrees from full extension. Players did two 5 s maximal voluntary contractions at each angle with 30 s rest between all repetitions. Then, players completed two submaximal repetitions and three maximal repetitions of eccentric knee extension at 60 degrees/s , with a 30 s break between the sets. We recorded the maximal torque (Nm) for each of the tests and report the results as the average of the right and left leg.

Countermovement jump height was measured on a force platform (HUR Labs, Kokkola, Finland) and calculated using the net impulse from the force-time curve. Players completed three attempts separated by 2 min breaks and jumped with hands on their hips and self-preferred kneeling depth. Only the highest jump height (cm) is reported.

40 m sprint was tested on an indoor running track. Wall-mounted photocells (Athletics Training System, IC Control Media and Sport, Bromma, Sweden) placed 1 m above the ground registered time every 10 m. Players started from a standing position with the front foot placed 30 cm behind the first photocells and had two trials separated by a 2 min break. We retained sprint times every 10 m (s) from the best trial for analysis.

Eccentric hamstring force was also assessed with the NordBord during training week 4 and 6, before a football training session, at the team training facility. Players had a short general warm-up and three submaximal repetitions before performing one set of three maximal repetitions without a weight vest.

All players and their coaches were asked to not perform any hard physical training the day before the pre- and posttests. At least two days separated the last training session with the Nordic hamstring exercise and the posttest.

### 2.4. Randomisation and Blinding

A person not involved in the assessment or training randomised the players with a 1 : 1 allocation within teams using a computer-generated list. All persons responsible for conducting the pre-, mid-, and post-tests were blinded to group allocation. The same equipment was used for all tests, and tests were performed by the same experienced testers on all occasions. The players, their coaches, and the researcher following up the Nordic hamstring training could not be blinded to group allocation.

### 2.5. Sample Size

From previous tests on all players in the Norwegian top division for women, we expected the average maximal eccentric hamstring force to be 300–350 N and the standard deviation to be 50 N. Based on previous studies, we expected a 30% increase in force (+100 N) in the high-volume group [24, 30] and 15% increase (+50 N) in the low-volume group [31, 32]. With a power of 80% and significance level at *p* < 0.05, a sample size of 16 participants per group was required to detect the expected between-group difference.

### 2.6. Statistical Analysis

All variables were tested for normality with the Shapiro–Wilk test. We used two-tailed paired *t*-tests to assess within-group differences between pre- and post-tests, ANCOVA (covariate: pretest results, fixed factor: group) for between-group differences in strength, jump, and sprint performance and unpaired *t*-tests for between-group differences in age, height, and mass. Differences in NordBord test results with 0 kg, 5 kg, and 10 kg added weight were analysed by repeated measures ANOVA. We imputed missing data (7% of values) from the NordBord tests in weeks 4 and 6 with the mean of the two closest tests and analysed strength over the four test occasions by split-plot ANOVA (within-factor: group, between-factor: time). We calculated the Pearson's correlation coefficient between the NordBord and the eccentric isokinetic dynamometer test. Compliance is expressed as the percentage of completed relative to assigned training sessions. Muscle soreness is the mean (±standard deviation) of all responses to the muscle soreness questionnaire. A *p* value of <0.05 was considered significant. A priori analyses were decided to be per protocol with compliance to the training intervention required to be ≥ 67%.

## 3. Results

### 3.1. Participant Flow

**Figure 2:**
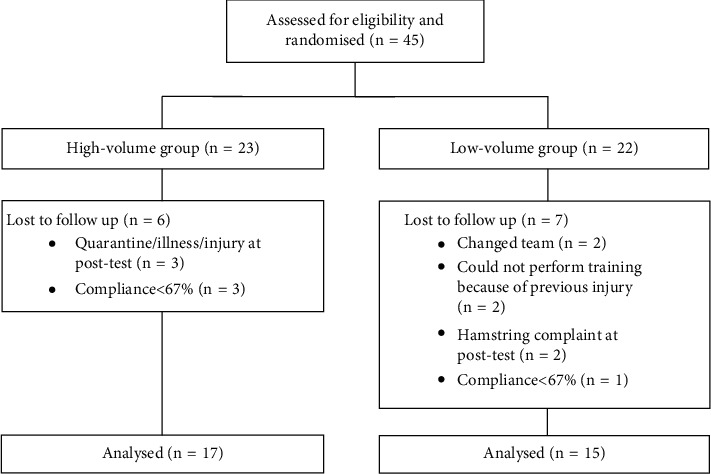
Chart showing flow of participants.

Thirty-two players completed the training intervention per protocol and were included in the analyses (Figure 2). The high-volume group completed 19 ± 2 of 21 planned Nordic hamstrings sessions (89%) and the low-volume group 9 ± 1 of 10 sessions (93%). Age (high volume: 21 ± 4 yrs, low volume: 20 ± 2 yrs, *p*=0.51), height (high volume: 167 ± 6 cm, low volume: 170 ± 5 cm, *p*=0.16), and body mass (high volume: 60 ± 6 kg, low volume: 64 ± 9 kg, *p*=0.11) did not differ between groups.

### 3.2. Maximal Eccentric Force

**Figure 3:**
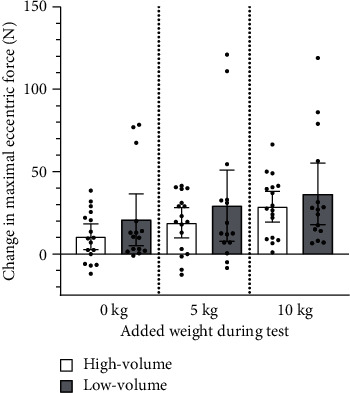
Change in maximal eccentric force for the high- (*N* = 17) and low-volume groups (*N* = 15) during NordBord testing with 0 kg, 5 kg, and 10 kg added weight. Results are presented as mean with 95% confidence intervals and individual values for change (•).

Both groups increased their maximal eccentric force in the NordBord tests—with no (high volume: 292 ± 52 to 303 ± 47 N, *p*=0.01, low volume: 296 ± 58 to 316 ± 46 N, *p*=0.01), 5 kg (high volume: 292 ± 56 to 311 ± 53 N, *p* < 0.001, low volume: 293 ± 67 to 322 ± 49 N, *p*=0.01), and 10 kg added weight (high volume: 294 ± 57 to 323 ± 58 N, *p* < 0.001, low volume: 293 ± 64 to 330 ± 51 N, *p*=0.001) (Figure 3). The increase in maximal eccentric force did not differ between groups in the NordBord tests, regardless of added weight (0 kg: *p*=0.11, 5 kg: *p*=0.25, and 10 kg: *p*=0.38).

Adding weight augmented the increase in maximal eccentric force from the pre- to the post-test (*p* < 0.001, partial eta squared = 0.47, observed power = 0.995). Pairwise comparisons showed that the increase was greater when tested with 10 kg added weight than with 5 kg (mean difference: 9 N, 95% CI: 1–16 N, *p*=0.018) or 0 kg (mean difference: 17 N, 95% CI: 9–25 N, *p* < 0.001) added weight.

When including the two intermediate NordBord tests without added weight in week 4 and week 6, there was a main effect for time on maximal eccentric strength (*p* < 0.001), but no interaction between group and time (*p*=0.52). Pairwise comparisons showed an increase in maximal eccentric force from the pretest to week 6 (mean change: 20 N, 95% CI: 8 to 31 N, *p* < 0.001) and to the posttest (mean change: 16 N, 95% CI: 5 to 27 N, *p*=0.002), but not to week 4 (mean change: 8 N, 95% CI: −2 to 18 N, *p*=0.22).

### 3.3. Maximal Eccentric and Isometric Torque

**Figure 4:**
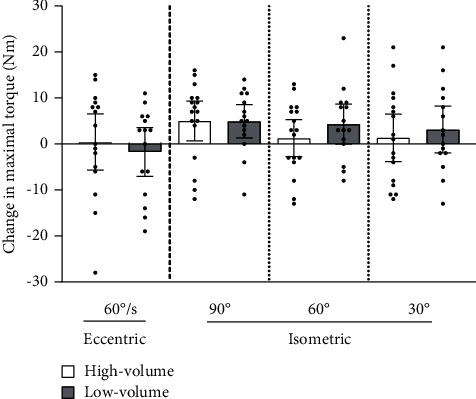
Changes in maximal eccentric torque at 60 degrees/s (*N* = 31) and maximal isometric torque at 90-degree, 60-degree, and 30-degree (*N* = 32) knee flexion from the pre- to the post-test for the high- and low-volume groups. Results are presented as mean of the right and left leg torque with 95% confidence intervals.

Maximal eccentric torque at 60 degrees/s did not change from the pre- to the post-test in either group (high volume: 118 ± 12 Nm to 118 ± 18, *p* = 0.88, low volume: 122 ± 21 to 121 ± 26 Nm, *p* = 0.54). Both groups improved isometric torque at 90-degree knee flexion (high volume: 64 ± 14 to 69 ± 13 Nm, *p* = 0.03, low volume: 63 ± 14 to 68 ± 15 Nm, *p* = 0.014), but not at 60-degree (high volume: 99 ± 12 to 100 ± 14 Nm, *p* = 0.55, low volume: 100 ± 13 to 104 ± 17 Nm, *p* = 0.06) or 30-degree knee flexion (high volume: 111 ± 13 to 113 ± 16 Nm, *p* = 0.57, low volume: 112 ± 15 to 115 ± 21 Nm, *p* = 0.20). We found no significant between-group differences in the change in maximal eccentric torque at 60 degrees/s (*p* = 0.52) or isometric torque at 90-degree (*p* = 0.86), 60-degree (*p* = 0.31), or 30-degree (*p* = 0.20) knee flexion (Figure 4).

### 3.4. Countermovement Jump and Sprint Performance

Countermovement jump height and 40 m sprint test results did not change from the pre- to the post-test in any of the groups, nor were there any differences between groups (Table 2).

### 3.5. Relationship between NordBord and Isokinetic Dynamometer Testing

**Figure 5:**
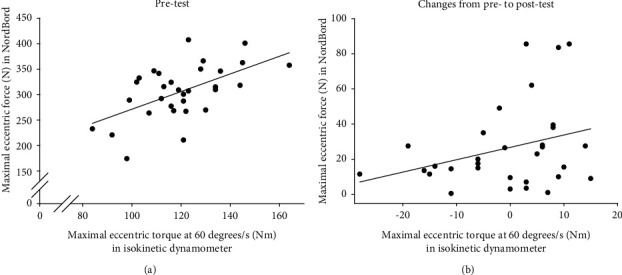
Scatter plot between maximal eccentric force in NordBord and maximal eccentric torque at 60 degrees/s tested in isokinetic dynamometer at pretest (a) and changes from pre- to post-test for the same tests (b). Results from both tests are average of right and left leg (*N* = 31).

We found a poor correlation (*r* = 0.56, *p* > 0.001) between maximal eccentric force in the NordBord test and maximal eccentric torque at 60 degrees/s tested in the isokinetic dynamometer at baseline, and the correlation (*r* = 0.31, *p* = 0.01) between the change from the pre- to the post-test for the two tests was even weaker (Figure 5).

### 3.6. Muscle Soreness

**Figure 6:**
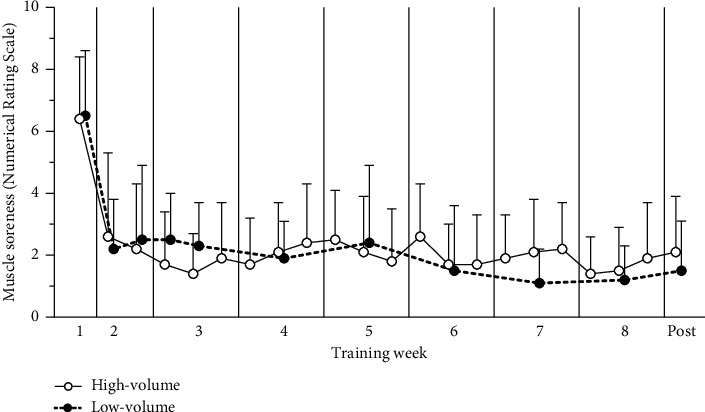
Hamstring muscle soreness reported on a numerical rating scale before all Nordic hamstring sessions during the intervention by the high- (○) and low-volume groups (•). Results are presented as mean and standard deviation.

Hamstring muscle soreness was reported to be mild throughout the entire training intervention period for both groups (high volume: 2.2 ± 1.7, low volume: 2.3 ± 1.7), except after the pretest (Figure 6). Response rates for the high- and low-volume group were 68% and 86%, respectively.

## 4. Discussion

This is the first study to compare the effects of high versus low training volumes of the Nordic hamstring exercise during the preseason in female football players. The main finding was that the evidence-based high-volume programme did not perform better than the low-volume programme at improving eccentric hamstring strength. Players had to train for at least 6 weeks to improve their strength. No change was observed in jumping or sprint performance.

Our main finding is in line with two previous studies that have compared different volumes of the Nordic hamstring exercise. Both among recreationally active men (440 vs. 128 total reps over 6 weeks) [24] and male elite youth football players (1 vs. 4 sets per week for 6 weeks) [31], there were no differences in strength adaptations in the groups that were compared. A meta-analysis on Nordic hamstring exercise training volume has also concluded that performing lower training volumes of the Nordic hamstring exercise does not attenuate adaptations in eccentric strength [33]. Conversely, football players performing the Nordic hamstring exercise twice per week for eight weeks increased their strength, while those training once per week did not [34].

Few studies have investigated the response to using the Nordic hamstring exercise among female football players, and only one has measured strength with a device that resembles the NordBord. Seventeen female elite players performing a high-volume Nordic hamstring programme during the preseason (8 weeks, 472 total reps) increased their hamstring strength by 13% [19], which is similar to the 10% and 13% increase we observed in our high- and low-volume groups, respectively. Our results are lower than the ∼20% increase found after Nordic hamstring interventions among male athletes [31, 35] and the *∼*30% increase seen among recreationally active men [24, 30]. Less concurrent training in the groups of recreationally active men can be one reason the latter studies observed greater strength gains, as concurrent training can attenuate improvements in strength [26]. Less experience with eccentric training, and therefore a greater potential for improvement, may be another reason.

Some players became strong enough to control the forward falling motion of the Nordic hamstring exercise throughout the range of motion. Without added weights, these may not have been able to reach their maximal eccentric force, since a prerequisite for the NordBord test is that players reach a critical point were the external gravitational force on the upper body exceeds their maximal eccentric hamstring strength [29]. This may explain that both groups had greater increase in maximal eccentric strength when the NordBord test was performed with added weights than without (Figure 3) and is the reason we emphasise the NordBord test results performed with 10 kg. We have only compared pre- and post-tests performed with the same weights.

Presland et al. [24] found muscular adaptations to happen early in the intervention with significant changes after only 14 days of training. We included mid-tests in training weeks 4 and 6 and found that strength did not increase significantly by week 4, but by week 6. In the systematic review by Cuthbert et al. [33], 4-week interventions found trivial to small differences in strength from pre to post, while 6–10 weeks saw moderate-to-very-large effect sizes.

Neither of our groups improved their isokinetic eccentric torque. This is in contrast to some previous studies [28, 36, 37], while other studies did not detect any change in isokinetic eccentric torque [38, 39]. The discrepancy between changes in strength measured with NordBord and isokinetic dynamometer may be explained by the low correlation we (Figure 5) and others find [40, 41]. Although both tests are designed to measure eccentric hamstring strength, they may measure different traits and be highly specific to the training mode chosen [42]. The NordBord test is very similar to the training exercise; this could be why we found improvements on the NordBord test but not on the dynamometer.

During isometric testing, it was surprising that we only detected an increase at 90-degree knee flexion, a knee angle where the lowest force is required when performing the Nordic hamstring exercise. We suspect this may be due to a lack of familiarisation to the test; isometric testing at 90-degree knee flexion was the first test performed. Therefore, it is possible that the increase is due to a learning effect. Another possibility is that players who were not able to control the forward falling motion through the full range of motion only trained in the first part of the movement. Including a version of the exercise that reduced the load for weaker player (e.g., using elastic bands) may have helped more players work at longer muscle lengths during the exercise.

One main challenge to facilitate player adherence with injury prevention is concerns over muscle soreness and “heavy legs” [43]. While unaccustomed eccentric exercise can cause muscle soreness, as we also observed after the initial pretesting session (Figure 6), a single session of eccentric training protects against muscle damage and soreness in subsequent sessions due to the repeated-bout-effect [44, 45]. We, like other studies using a careful, gradual increase in training load [28, 30], found the Nordic hamstring exercise to cause low levels of muscle soreness throughout the training intervention. Still, it should be noted that some players reported persistent soreness throughout the entire training period. Performance enhancing effects of an injury prevention programme may increase buy-in from players and coaches. Previous studies have indicated the Nordic hamstrings exercise can improve sprint, jump height, repeated sprint, and acceleration [46–48]. In our study, however, neither group had any changes in jump height or sprint times.

### 4.1. Limitations

We were unable to perform familiarisation sessions before the pretests. We have previously tested 21 female top-division players one week apart without familiarisation and found good test-retest reliability for NordBord (unpublished data: 95% limits of agreement: −8 N (−32 N; 17 N), intraclass correlation coefficient: 0.93 (0.80–0.97), standard error of the mean: 11 N (3%)), and isokinetic eccentric torque, jump height, and sprint speed did not change from pre- to post-testing. Therefore, we consider it unlikely that there was a learning effect. One of the teams was put in quarantine for a week because a player tested positive for COVID-19 (no one else was infected). During that week, these players performed the Nordic hamstring programme on their own and we followed up their training via phone. As players were randomised within teams, this should not cause any systematic error. As we do not have a control group, the improvements in strength can potentially be caused by football training and not the Nordic hamstrings training. We consider this unlikely, as amateur players tested 8 weeks apart did not change their eccentric hamstring force, when training football only [19]. However, football training may have contributed to some of the reported muscle soreness. We did not include a control group because we considered it unethical to deny football players from taking part in an evidence-based injury prevention programme, and it was not necessary to answer our primary research question. Because some players were lost to follow-up (Figure 2), the number of players completing the intervention in the low-volume group was slightly below our sample size calculations. This reduces our statistical power.

## 5. Conclusion

Female football players performing a high-volume Nordic hamstring programme during the preseason did not increase hamstring strength to a greater extent compared to players performing a low-volume programme. Both groups increased their maximal eccentric force measured with the NordBord, and we observed significant improvements after 6 weeks of training. Neither group improved their maximal eccentric torque measured by isokinetic dynamometer. The poor correlation between the two strength tests may explain this difference. None of the programmes improved jump height or sprint performance.

### 5.1. Perspectives

Our findings demonstrate that a low-volume Nordic hamstring exercise programme is equally effective as the evidence-based high-volume programme in increasing eccentric hamstring strength among female elite football players. Performing a low-volume programme is probably more feasible and likely to be adopted in a real-world context than a high-volume programme; as such, our findings have important implications for future implementation of the Nordic hamstring exercise among female elite football players.

## Figures and Tables

**Table 1:** Training protocol for the high- and low-volume training groups.

	High-volume group		Low-volume group	
Week	Sessions	Sets and repetitions	Sessions	Sets and repetitions
1	1	2 × 5	1	2 × 4
2	2	2 × 6	2	4 × 6
3	3	3 × 6–8	2	4 × 6
4	3	3 × 8–10	1	2 × 4
5	3	3 × 12-10-8	1	2 × 4
6	3	3 × 12-10-8	1	2 × 4
7	3	3 × 12-10-8	1	2 × 4
8	3	3 × 12-10-8	1	2 × 4

Total	21	538	10	144

**Table 2:** Pre- and post-test results in the countermovement jump test and 40 m sprint for the high- (*N* = 16) and low-volume groups (*N* = 14). Two participants are missing because they did not perform the test or there was a measurement error. Results are presented as mean ± standard deviation.

	High-volume group			Low-volume group			Between-groups *p* value
	Pre	Post	*p* value	Pre	Post	*p* value	
Countermovement jump							
Jump height (cm)	31.9 ± 5.7	31.1 ± 5.9	0.08	29.4 ± 5.0	29.6 ± 5.2	0.69	0.20
Sprint							
10 m (s)	2.01 ± 0.06	2.03 ± 0.08	0.31	2.05 ± 0.12	2.06 ± 0.14	0.71	0.66
20 m (s)	3.44 ± 0.11	3.45 ± 0.13	0.45	3.52 ± 0.21	3.54 ± 0.23	0.52	0.99
30 m (s)	4.79 ± 0.16	4.80 ± 0.18	0.85	4.93 ± 0.30	4.96 ± 0.33	0.46	0.67
40 m (s)	6.15 ± 0.22	6.15 ± 0.24	0.85	6.35 ± 0.41	6.37 ± 0.44	0.65	0.65

## Data Availability

The data used to support the findings of this study are available from the corresponding author upon request to roara@nih.no.
